# Adaptive Molecular Evolution of *AKT3* Gene for Positive Diversifying Selection in Mammals

**DOI:** 10.1155/2020/2584627

**Published:** 2020-05-19

**Authors:** Farmanullah, Seyed Mahdi Hosseini, Aixin Liang, Guohua Hua, Zia ur Rehman, Hira Sajjad Talpur, Mohammad Salim, Saeed Ahmad, Adili Abulaiti, Momen Khan, Muhammad Safdar, Ihsan Ullah Kakar, Zahoor Ahmad, Muhammad Zulfiqar Ahmad, Ye Tingzhu, Nicola M. Schreurs, Iqra Bano, Liguo Yang

**Affiliations:** ^1^Faculty of Veterinary and Animal Sciences, Department of Livestock Production, Lasbela University of Agriculture, Water and Marine Sciences, Uthal, Pakistan; ^2^Key Laboratory of Animal Genetics, Breeding and Reproduction, Ministry of Education, College of Animal Science and Technology, Huazhong Agricultural University, Wuhan, Hubei 430070, China; ^3^Department of Animal Health, Faculty of Animal Husbandry and Veterinary Sciences, The University of Agriculture Peshawar, Pakistan; ^4^Department of Animal Breeding and Genetics, Sindh Agriculture University, Tandojam, Pakistan; ^5^Department of Forestry and Wildlife Management, The University of Haripur, Khyber Pakhtunkhwa, Pakistan; ^6^Institute of Biological Sciences, Sarhad University of Science and Information Technology, Peshawar, Khyber Pakhtunkhwa, Pakistan; ^7^Livestock and Dairy Development, Khyber Pakhtunkhwa, Pakistan; ^8^Department of CMS (FVAS), Lasbela University of Agriculture, Water and Marine Sciences, Uthal, Balochistan, Pakistan; ^9^Adaptive Research Program, Quetta, Pakistan; ^10^Guangdong Provincial Key Laboratory of Plant Molecular Breeding, Guangdong Subcenter of National Center for Soybean Improvement, College of Agriculture, South China Agricultural University, Guangzhou 510642, Guangdong, China; ^11^Animal Science, School of Agriculture and Environment, Massey University, Palmerston North, New Zealand; ^12^Key Laboratory of Henan Province for Drug Quality and Evaluation, Ministry of Education of China, School of Pharmaceutical Sciences, Zhengzhou University, Zhengzhou, China

## Abstract

The V-Akt Murine Thymoma Viral Oncogene Homolog 3 (AKT3) gene is of the serine/threonine-protein kinase family and influences the production of milk fats and cholesterol by acting on the sterol administrative area restricting protein (SREBP). The AKT3 gene is highly preserved in animals, and during lactation in cattle, its expression increases. The AKT3 gene is expressed in the digestive system, mammary gland, and immune cells. A phylogenetic investigation was performed to clarify the evolutionary role of AKT3, by maximum probability. The AKT3 gene sequence data of various mammalian species was evident even with animals undergoing breeding selection. From 39 mammalian species studied, there was a signal of positive diversifying selection with Hominidae at 13Q, 16G, 23R, 24P, 121P, 294K, 327V, 376L, 397K, 445T, and 471F among other codon sites of the AKT3 gene. These sites were codes for amino acids such as arginine, proline, lysine, and leucine indicating major roles for the function of immunological proteins, and in particular, the study highlighted the importance of changes in gene expression of AKT3 on immunity.

## 1. Introduction

An evolutionary study provides an understanding of the genetic development occurring across species. Often a solitary ancestor is responsible for the initiation genetic variation within a population. Speciation, common descent, and natural selection are the main features of evolutionary process. This is understood and explained by various branches of biological sciences including genetics, paleontology, and ecology. Recently, research has become more focused on understanding the evolutionary process of life during the various phases of evolution. In particular, the research is concerned with evaluating genetic diversity, understanding the heritability of important traits, reasons for the molecular evolution, and the ability of genes to contribute through breeding selection, biogeography, and genetic drift.

The field of evolutionary research stimulates research into the evolution of cooperation, ageing evolvability [1], speciation [2], and sexual reproduction [3]. Evolutionary biology helps us to understand gene function and the processes of genetic variation and gene transfer including the study of point mutations, gene and genomic duplication, heritability rates, probability, and genome-wide associations [4]. The focus of molecular evolution is to consider those genes that are associated with advantageous traits and their ability to be disseminated through a population via selective breeding [5].

The AKT family of genes has a role in mammary gland growth, lactation, and mammary degradation, and their isoforms are candidate genes for milk production [6]. The AKT gene family is involved in a diversity of genetic processes including cell propagation, differentiation, angiogenesis, apoptosis, tumor genesis, metabolism, cell endurance, development, glycogen synthesis, and glucose uptake [7, 8].

Mammalian cells contain three genes that encode for three isoforms of AKT, namely, AKT1 (PKB*α*), AKT2 (PKB*β*), and AKT3 (PKB*γ*). All Akt isoforms contain a N-terminal administrative pleckstrin homology (PH) area, a focal kinase domain with serine/threonine explicitness, and a C-terminal hydrophobic space [9]. The AKT3 gene is a constituent of the serine/threonine protein kinase family and has a function in controlling fat and cholesterol composition in the milk by modifying the action of the sterol administrative component restricting protein. The expression of AKT3 is highly variable in mammals. The AKT3 is highly expressed in the digestive system followed by the mammary organ and is also expressed in immune cells. It is associated with the TLR pathways as adequately as proinflammatory cytokines [10]. AKT3 is highly expressed in immune cells and contributes to immunity processes [11]. During lactation in cattle, the expression levels of AKT3 were increased [6].

The expression of AKT3 is found in low levels all through the human body [12], but AKT3 is the least measured isoform. However, the AKT3 gene has a putative oncogenic function given that is overexpressed when there is high enzymatic action in the endoplasmic reticulum of malignant breast cells [13]. The ongoing recognizable proof of somatic mutations of AKT3 including MAGI3-Akt3 and Akt3E17K in various malignancies likewise focuses on the significant role of this isoform in tumorigenesis [14]. AKT3 was the most enhanced isoform in numerous malignant growths, cancer, including GBM, ovarian, melanoma, endometrial, and breast cancer (O'Hurley et al., 2014; [13]).

Positive selection produces variation in phenotypes among animals and is a mechanism for disseminating favorable genes in a population. The goal of this study was to investigate and determine selection markers utilizing a maximum likelihood probability approach for the distinction of molecular genetics of AKT3 among mammalian species and provide information regarding the applicability of marker assist selection in the diverse species.

## 2. Material and Methods

### 2.1. Dataset Preparation and Sequence Analysis

Publically available gene banks such as Ensembl (http://useast.ensembl.org/index.html), NCBI (http://www.ncbi.nlm.nih.gov/genbank), and UniProt (http://www.uniprot.org) were considered, but the NCBI database was used for coding the nucleotide and amino acid sequence of *AKT3* for recovery and data analysis. The alignment of the sequences was performed with the help of Clustal Omega, in the MEGA 6.0 program [15]. Maximum likelihood methods were used to devise the phylogenetic tree within MEGA 6.0 for the *AKT3* gene. Bootstrapping provided 1000 replicates for the clustering of taxa. The log likelihood of the topology and branch length indicated the number of substitutions per site [16, 17]. The species were identified by their accession number and their mRNA and protein accession numbers as listed in Table 1. The NCBI gene bank accession numbers for the mammalian gene AKT3 datasets were used for testing our hypothesis to construct various datasets.

### 2.2. Analysis on the Bases of Codon Core Positive Selection

The present study was designed to study and investigate the molecular basis of evolution and the effect of positive selection of *AKT3* by analyzing the codon and sequence of AKT3 and comparing the dN/dS ratio of *ω* for two maximum likelihood approaches [18, 19]. Software tools utilised were DATAMONKEY (http://www.datamonkey.org/) in conjunction with the HyPhy package [20].

The analyses are completed in two steps. The 1st step was to find out the maximum likelihood ratio test for positive selection, where *ω* > 1 indicates the sites of expression. Two models were represented in each analysis by comparing its different sites with *ω* > 1 are (null), while the other is discrete general [16, 21] where the distribution of X^2^ compared with DF = 4 is the likelihood log (2*Δ*1). When the M7 model (null) is used, the interval is assumed to be between 0 and 1 with *ω* restricted and a *β* distribution. The alternative was a M8 model where the *ω* value is greater than 1 and obtained from the dataset. The rates of synonymous and nonsynonymous variation were used to calculate and identify positive selection of the *AKT3* gene. Using the fixed effect tests of different likelihood of sequences aligned for each site, Ahmad et al. [21] reported the various likelihood programs as random effect likelihood (REL), single likelihood ancestor counting methods (SLAC), and FEL likelihood, to approximately investigate the values globally of *ω*. Only those sites common in all tools were selected. The REL used 95% confidence interval for positive site selections perceived and used Bayes factor values > 20. The other analysis measured values of significance was *p* values < 0.05. Many sites were identified at *p* < 0.05 in different genes by the various software platforms.

The second step was to use likelihood tests to confirm the amino acid availability. Bielawski and Yang [22] reported that *ω* for different classes were used to estimate and investigate for each site of the posterior probabilities inferred by using Bayes theorem. The amino acid residues considered as being under selective pressure had a higher value and probabilities of *ω* > 1. Kelley and Sternberg [23] reported the Phyre and Swiss models (http://www.sbg.bio.ic.ac.uk/phyre2/html and http://swissmodel.expasy.org), and positive selection was assessed by amino acid location using crystalline structure. Glaser et al. [24] reported the bioinformatics tools that were used to predict and find out the protein in which the conserved evolutionary amino acid/nucleic acid level and position are found and on which the sequence phylogenetic relationships were based. Also, the ConSurf server link (http://consurftest.tau.ac.il) was used to predict and find the proteins in which amino acids and nucleic acids had been conserved through evolution (reference). The selection pressure was used to identify important codon sites. The Selection version 2.2 (http://selecton.tau.ac.il/) was used for sequence codon alignment of *AKT3*. Yang et al. [25] reported that Bayesian inference methods supported by maximum likelihood test were used accurately to measure the *ω* ratios of various codon-aligned sequence shifting.

### 2.3. Analysis of Protein-Protein Interaction Network

Analysis of the protein-protein interaction network is also crucial for further understanding of the *AKT3* molecular function. Gene interactions with *AKT3* were predicted using the special linkage analysis of STRING (version 9.1, http://www.string-db.org/) [26]. The web server data bank of biological interactions was used for the identification and identification of interactions of proteins. The cutoff standard value was used as the pooled score < 0.4. The highly connected and essential biological function proteins were indicated in the middle nodes. These were identified, documented, and estimated by the number of line connections between proteins of each node and using the resemblance value. Various software and tools were used for protein- protein interaction. The STRING and Cytoscape software tools were used for network construction and visualization of proteins and interactions [27].

### 2.4. Phylogenetic Tree of *AKT3*

The phylogenetic tree for the *AKT3* gene was constructed for thirty-nine species. The nucleotides of these various species were downloaded from the public database of NCBI for construction of phylogenetic tree. We have used MEGA 6 for phylogenetic tree construction after aligning the sequence of these species in Clustal w.

### 2.5. Domain Sites for AKT3

The candidate gene for mastitis-associated *AKT3* is a family member of the serine/threonine protein kinase family. Milk fat synthesis and cholesterol is the main function, regulated by sterol regulatory element binding protein (SREBP). We have searched the domains with InterPro Scan in EBI [28]. In our present integrated study of the evolution of the *AKT3* gene, a further approach is to search and find out the domains in mammals with InterPro Scan [28] using the search tool.

## 3. Results and Discussion

The present advances in the crucial record of genetic contrast have anticipated account recommendations for investigation of the positive selection objectives, which in the end would be significant to illuminate the hereditary suggest and choice work in evolutionary components. In addition, positive choice marks hinder the genomic areas that assume noteworthy jobs. Therefore, investigating such areas will give extensive help to recognizable proof of hereditary deviations, which would encourage the interruption of these utilitarian districts and movement in phenotypic combinations. The enlightenments of the hereditary bases of various characteristics in many species have been premeditated by competitor gene methodology. The recognizable proof of these candidate genes assumes a significant role in phenotypic variety in domesticated animals' populace and gives new development in the evolutionary procedure and positive choice (Brown et al., 2013).

**Figure 1:**
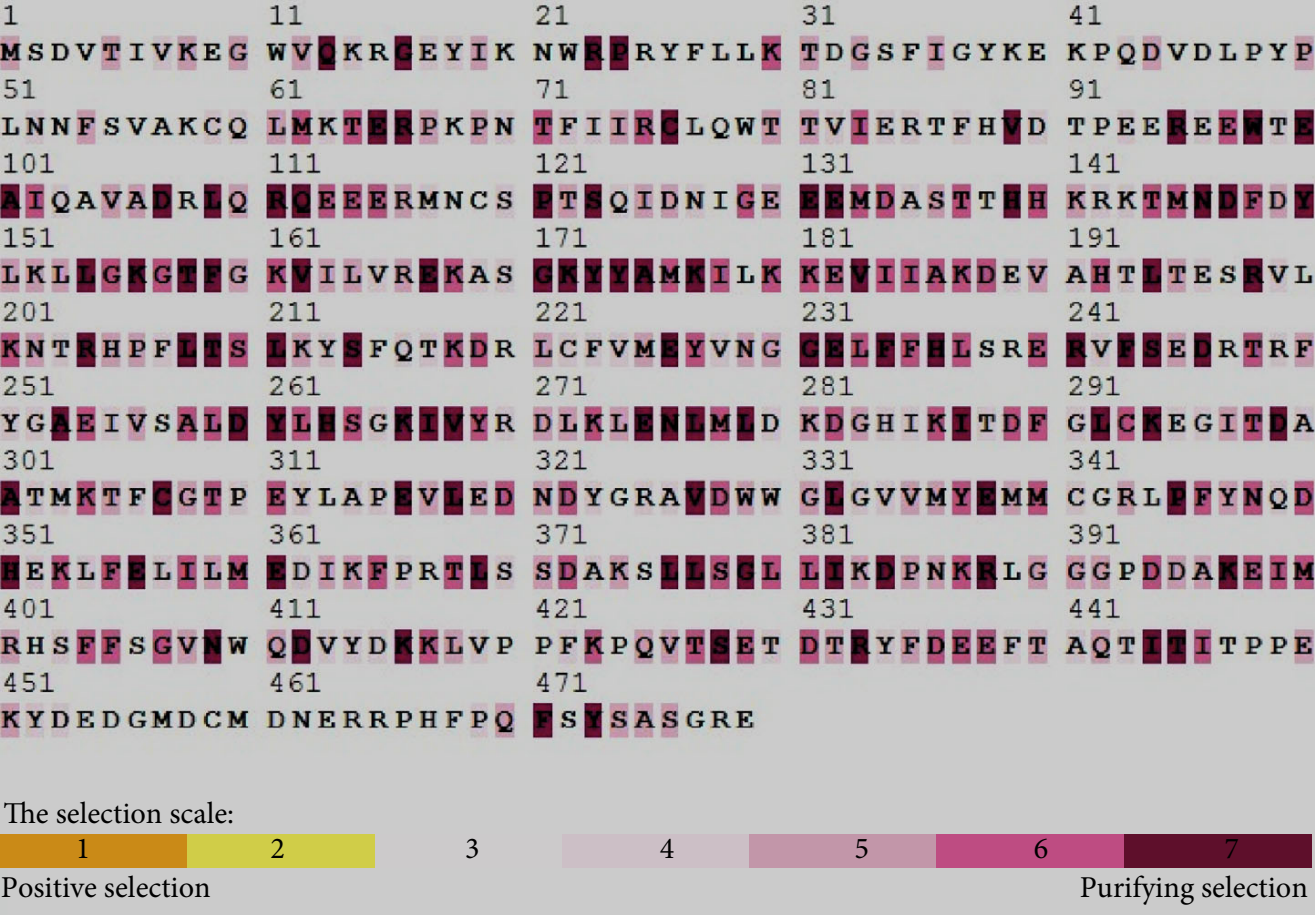
The selection tool model of mechanistic empirical combination (MEC) was used for selection pressure of mammal *AKT3* gene sequences. Positive selection was represented by yellow and brown highlights purifying selection; neutral selection was represented by gray and white highlights, while negative selections on codons were represented by purple color highlights.

The *AKT3* lineage was <1 for the average *ω* across the site ratio (dN/dS). This indicates that, based on the similarities between sequences on the phylogenic relationship, there was many conserved amino acid even though positive selection had occurred. The indicators of selection were masked by the large number of conserved amino acids; however, many amino acids were found to be positively selected. Selection results are shown with color scales in Figure 1. From the likelihood approaches used in this study, there were 14 codon *AKT3* sites amenable to positive selection. Codon position of the positive selection sites for AKT3 was detected using REL which discovered four sites, FUBAR identified fourteen sites, and MEME exposed ten sites (Table 2). The number of positive selection sites for *AKT3*, using REL, FUBAR, MEME, and IFEL, was 33, 418, 20, and 1, respectively.

### 3.1. Position of Amino Acid and Positive Selection

**Figure 2:**
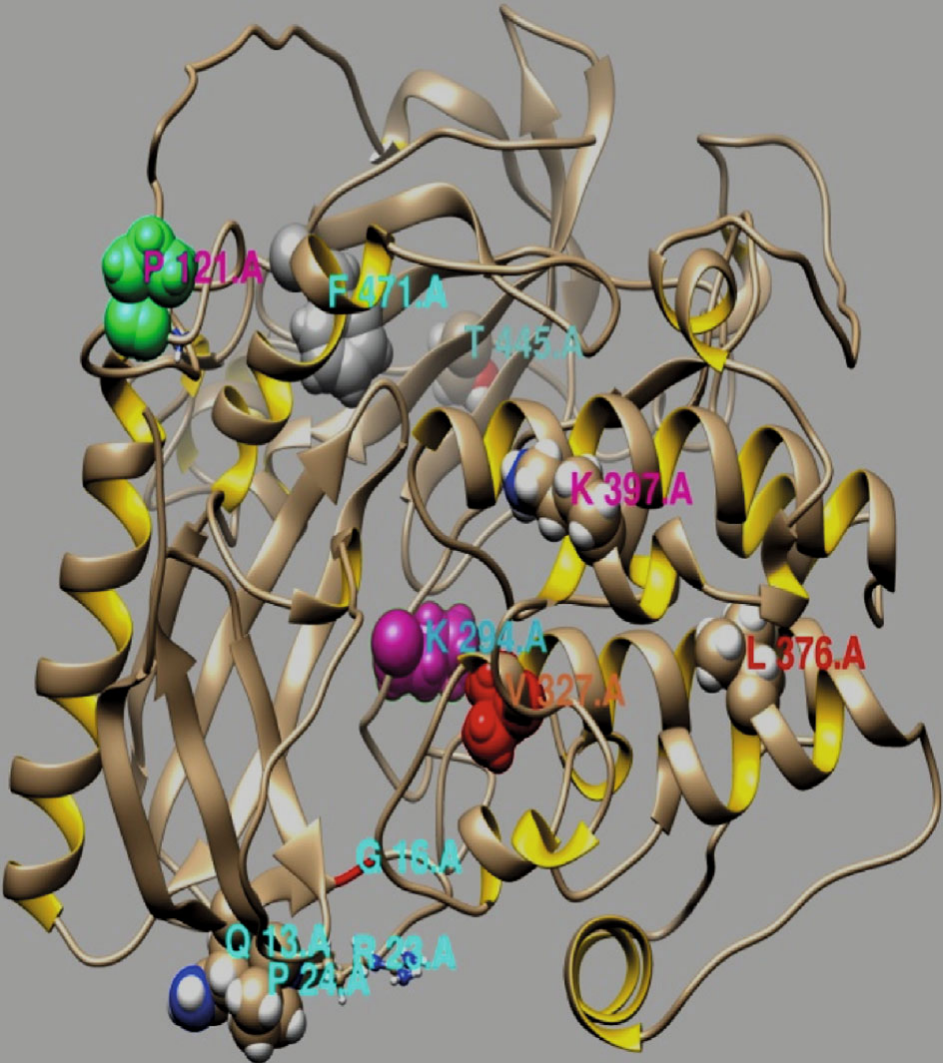
The *AKT3* gene sites of positive selected amino acids and location. The Phyre tool (http://www.sbg.bio.ic.ac.uk/phyre2/html) was used to construct the crystal structure of the positive selected sites. The crystal structure of bovine AKT3 as a reference; positively selected sites were drawn onto the crystal structure. All the residues which fall in the domain of the binding ligand sites, identified as under the positive selection. Both types of the site as ligand binding and signal sequence. The ligand binding sites were defined as those which fall in the region, while another cluster immediately following the signal sequence in the region.

**Figure 3:**
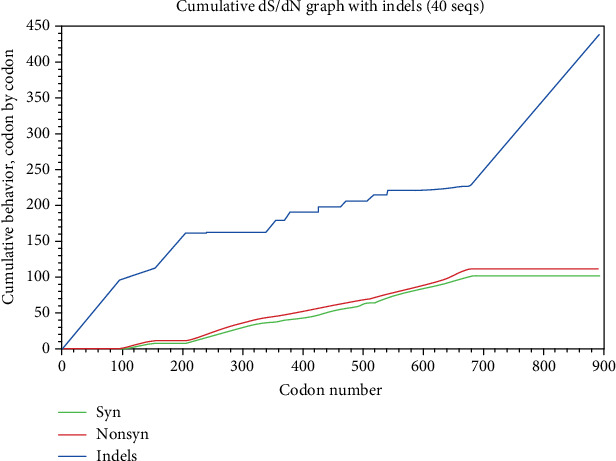
Cumulative behavior of synonymous, nonsynonymous, and ambiguous codon mutation changes per site comparing the mutation change estimates between the number of synonymous, nonsynonymous mutations and idels (ambiguous codons) per site; red plot indicating nonsynonymous, green synonymous, and blue for ambiguous codon positions.

The structure and function of protein is important for its continuity. Consequently, the sites that were detected as being positively selected may instruct and clarify the *AKT3* gene function. Using the crystalline structure of bovine AKT3 as a reference, the positively selected sites were elucidated. The high-probability sites were expected to be important for positive selection with *ω* > 1. The location, positive selection sites, and amino acid position were shown in Figure 2. The collective performance of identified codon locations was plotted (Figure 3), and the collective performance of ambiguous, synonymous, and nonsynonymous codon deviations with evolutionary time unit was represented. For the number of starting codons, the collective performance of the synonym mutation is decreasing and then amplified with the evolution of codons, while the performance of nonsynonymous codons is increasing with the passage of evolutionary time unit according to codon position, but it is lower in the position initially and then gradually increases. The ambiguous codons' performance increases by starting codons' position and then becomes constant.

### 3.2. Codon Model Selection

**Figure 4:**
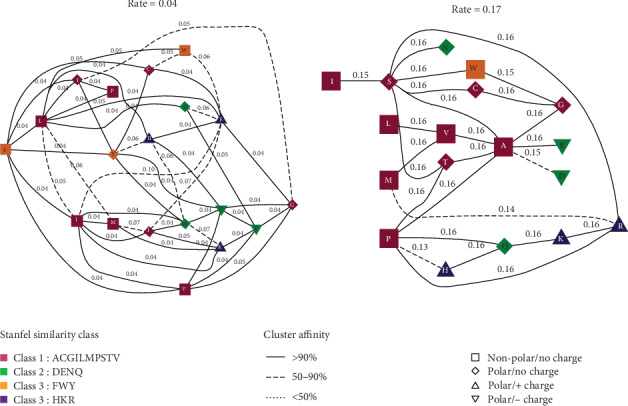
The use of a genetic algorithm (GA) model for identification of structure and evolutionary rate cluster was from AKT3 gene alignment of different species. Every cluster is labeled with maximum likelihood estimation, and its rate is concluded by GA. The nodes (residues) are interpreted by their Stanfel class and biochemical characteristics, and the edges (rate) are marked with the average rate estimation of the GA model.

We further analyzed the selection pattern derived by the evolutionary selection forces on amino acid sites in AKT3 proteins. We used different codon models available in DATAMONKEY web server. We found that there was adaptive evolution in basic amino acid sites in these proteins with different substitution ratios during evolution. The maximum substitution rate was 0.17 for the different ratio classes, and the minimum was 0.04 among various amino acid sites in AKT3 genes (Figure 4).

The nucleotide and amino acid substitution in the codon model was used to identify the synonymous and nonsynonymous substitutions [29], and the substitution model was used to confirm the significant change rate in nucleotides over amino acid position [30, 31]. The codon model of evolution using the genetic algorithm was used to identify the evolutionary fingerprinting in the coding sites of AKT3 genes. The codon model of evolution [20, 32, 33] used phylogenetic Markov model that includes substitution rates, character frequencies [34], amino acid substitution rate clustering [35, 36], and branch lengths through maximum likelihood estimation method. This resulted in 8245 models that were used in codon model selection based on the likelihood log and modified Bayesian Information Criterion (mBIC).

The selective effects associated with an exchangeable preference for particular amino acids were found in the AKT3 genes with a model that used the combined empirical codon and transition/transversion-related physicochemical parameters [37, 38]. The model with log (L) value -12865.8 for AKT3 was considered the best for amino acid substitution analysis. We have observed the mBIC values 27550.74 two class rates for the distribution of amino acids in different classes (Figure 4) with an estimation of single rate *dN/dS* substitution (Table 3). The genetic algorithm multirate model was used to analyze the class rates to calculate the substitution rate at the amino acid level during the evolutionary time scale (Figure 4). The substitution rate in each class was calculated through genetic algorithm models by using the Stanfel class parameters [36]. The substitution rate distributed the amino acids into three classes through the evolutionary rate cluster and the substitution pair FWY and HKR have <50% substitution, DENQ has 50% substitution, and ACGILMPSTV has substitution rate 90%.

### 3.3. Network of Protein-Protein Interaction

**Figure 5:**
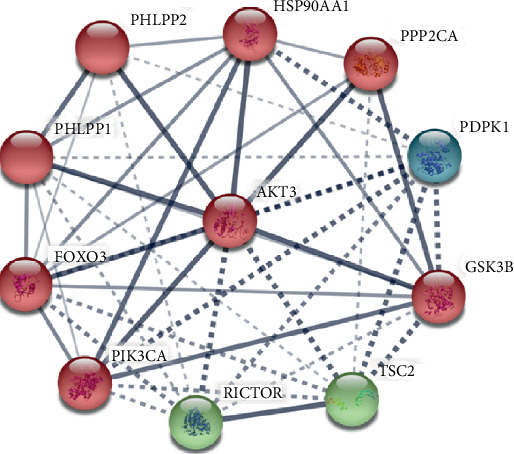
The protein-protein interaction (PPI) network was built by STRING database for AKT3 genes. Gray and red circles characterize downregulated and upregulated genes, respectively. Line thickness indicates the strength of the interaction. Dash and solid edges mean negative and positive correlation coefficient. Network nodes denote proteins' posttranscriptional modifications or splice isoforms, and each node represents all the proteins produced by a single, protein-coding gene locus.

We have used the STRING data bank to search the encoded protein of *AKT3* and found numerous PPI pairs. The PPI predicted network had 31 nodes (denoted by *AKT3* encoded proteins) and 308 edges (the line networks between nodes) as shown in Figure 5. The value of the average local clustering coefficient is 0.887. The *p* value of PPI enrichment is 5.33*e*-11. Ten genes that are coexpressed genes in the PPI network and showing an interaction with AKT3 are as follows: *RICTOR*, *TSC2*, *GSK3B*, *PDPK1*, *PPP2CA*, *HSP90AA1*, *PHLPP2*, *PHLPP1*, *FOXO3*, and *PIK3CA* (Figure 5). The Human AKT3 sequence was used as a reference for the PPI network analysis. These genes may be involved in biological signaling pathways due to the upregulation of AKT3 [39].

### 3.4. Phylogenetic Tree of AKT3

**Figure 6:**
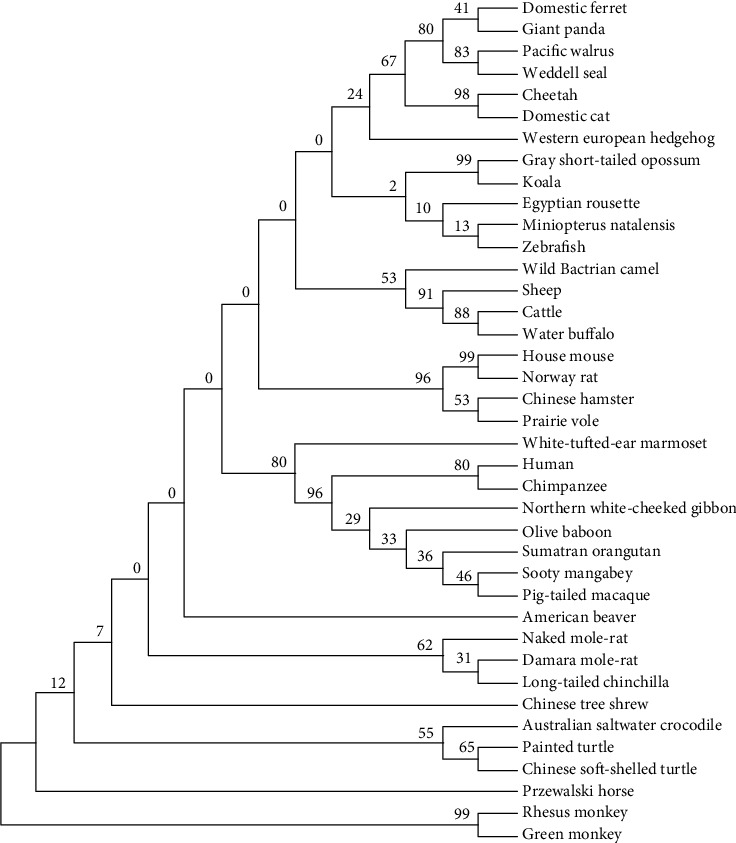
Phylogenetic tree constructed using the *AKT3* gene full length proteins of various 39 species. The neighbor-joining method was used for the multiple alignments with the help of MEGA 6.0 software.

The NCBI database was used to download the coding sequence and protein sequences for construction of the phylogenetic tree. Using the MEGA 6 Clustal W [15] for aligned sequences, a phylogenetic tree was constructed as shown in Figure 6. In the tree, it is shown that the genes which are evolutionally close form groups with various species with less significant relationships making up different groups.

**Figure 7:**
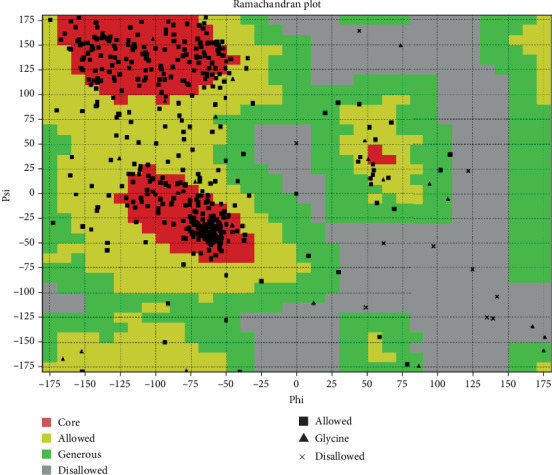
Ramachandran plot prediction to examine the structure of a protein, the conformation of the amino acid present in the protein, and close associates between the atoms.

We have also applied the Ramachandran plot to predict for the *AKT3* using http://vadar.wishartlab.com/. A Ramachandran plot is used to visualize energetically allowed regions for a polypeptide backbone torsion angle psi (*ψ*) against phi (*φ*) of amino acid residues present in a protein structure. The main chain N-Calpha and Calpha-C bond of the polypeptide of the Ramachandran plot has free rotation. The relative rotational angle of torsion was represented by phi and psi, respectively. In nature, the peptide bond is rigid and planar. To understand the Ramachandran, it is important to have knowledge of peptide bond structure. The analysis of the protein structure and the key role played by some amino acids and close contacts of the atoms in protein is shown in Figure 7.

### 3.5. Domains for the *AKT3*

**Figure 8:**
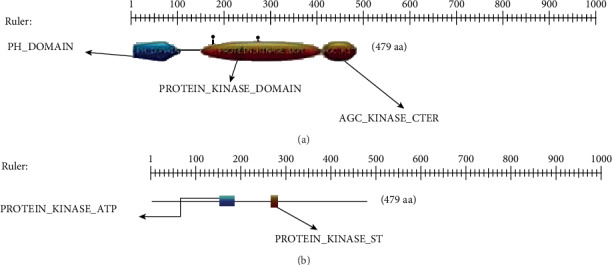
The *AKT3* visualization domain results in Bos taurus. (a) The domain hits were by profiles of the *AKT3* in Bos taurus. (b) The domain presentation hits by patterns in *AKT3* of Bos taurus.

The visualization domain results are shown in Figure 8, and each domain information was given in Table 4, as domain table number.

AKT3 inadequacy did not influence macrophage apoptosis; however, it advanced macrophage cholesterol collection, lipoprotein uptake, and foam cell development in vitro, through balancing out Acetyl-Coenzyme A acetyltransferase 1 (ACAT1) [40]. Another investigation found that knockdown of AKT3 prompted diminished bad phosphorylation and expanded caspase-9 and caspase-3 action, indicating that these isoforms are significant for cell suitability through guideline of mitochondrial layer potential [41]. The AKT3 gene showed an interesting phenotype of providing anchorage haven independent development and invasion [42]. The AKT3 signaling pathway is constitutively dynamic in ~70% of advanced-phase organize melanomas, and segments of this pathway correspond to potential therapeutic targets since it assumes a significant function in melanoma development, to a limited extent, by suppressing the cell bond particle E-cadherin [43]. While AKT3 substrates associated with mediating its effect on proliferation, apoptosis, and chemoresistance in melanoma have been distinguished, their observation is the first to uncover an immediate substrate concerned with AKT3-induced melanoma relocation [44].

AKT3 oversees glioma succession and restorative resistance by means of initiating DNA twofold strand break fixation. AKT3 is dominating in the core of glioma cells, and the mark genes related with AKT3-driven tumors are concerned with the DNA fix pathway (HR: homologous recombination; NHEJ: nonhomologous end joining) [45]. The AKT3 signaling pathway assumes a basic function in melanoma arrangement and invasion, and segments of this signaling cascade are along these lines deserving of focus for the treatment of harmful melanoma [44]. They have distinguished the AKT3 target site at serine residue 720 in the TBX3 protein and show that this site is phosphorylated in vivo. This discovery involves AKT3 as a positive controller of TBX3 protein security. AKT3 phosphorylates TBX3 at serine 720 (S720) and improves TBX3 protein solidity (Jade Peres et al., 2014). Phosphorylation by AKT3 advances TBX3 atomic confinement and transcriptional restraint of E-cadherin [44]. Susceptibility to experimentally induced autoimmune system encephalomyelitis was found to be controlled and regulated by *AKT3* via the central sensory nervous system and insusceptibility framework. This is because the appropriate function of CNS cells and the management and regulation of T cell capacity require the presence of *AKT3*.

The past investigations demonstrate that the isoform *AKT3* of the AKT family is associated with assorted functionality. She [46] reported the gene evolution, phylogenetical branch length, and positive selection analysis study on *AKT3*, and they used the 39 nucleotide coding sequences of various species of mammalian species to investigate the selection pressure experienced. To perform these evaluations using the proregion from *AKT3*, mature and complete sequences were used. In these mammalian clades, we have found the positive selection codon sites, by study of the phylogenetic tree. We identified 32 positive selection sites with REL, 414+4 sites with FUBAR, 20 sites with MEME, and 1 site with IFEL and selected those which are common to all analyses. We have measured the adaptive selection pressure at codons of the *AKT3* sequence and used the mechanistic empirical combination (MEC) model in selection serving to promote positive selection. Axelsson et al. [47] reported that the dN/dS ratio might increase through conversion of genes with GC nucleotide pairings. Analyses using Empirical Bayes investigated the position of amino acids for the *AKT3* gene. The positive selection signals were found at multiple codon positions, and it showed that the selection that will take place on these positions across decades on these selected sites will play key role in signaling. Auclair et al. [48] reported and detected selection positive signals among 24 mammalian species in the human BMP15. Hence, under positive selection, amino acids sites are important for protein specialty function [49]. They further [49] reported that BMP15 evolved and allowed positive selection faster in TGF family members in mammalian clade. In this study, we found the positive selection was in *AKT3* gene with *ω* > 1. The result showed synonymous and nonsynonymous (dN) sites and indicated the quicker and more evolved sites of nonsynonymous and new variants favored by and following in the balancing/purifying selection influenced by positive selection [50]. The protein structure validation and alteration allow identification of changes that affect the signaling pathway [51]. The common lineage divergence discrete result might be the species across substitution amino acid changes and settles with anterior submission. Scannell et al. [52] reported that the evolutionary routes from common ancestors differ for recent orthologs, which on homologous sites in selected lines may have resulted in genetic deviation. Therefore, for understanding mammalian genomes, the study of selection might stimulate potential exploration areas in the future.

## 4. Conclusion

In summary, we have found the selection pressure in mammalian species clade that *AKT3* has evolved swiftly. We have used a series of analysis for evolution study in *AKT3* using 39 various species coding sequences. We have found various positive selections sites in the gene under study. These positive sites of selection will be important for further study for their role in protein function. This *AKT3* gene selection analyses will help and assist in the development of breeding strategies to emphasize advantageous traits.

## Figures and Tables

**Table 1:** List of species and accession number of the NCBI gene bank database, which was used for the hypothesis testing.

S. No.	Species	Accession number	mRNA accession number	Protein accession number
1	Human	NM_005465.4	NM_006642.5	NP_859029.1
2	House mouse	NM_001357390.1	XM_030253917.1	XP_030109770.1
3	Norway rat	XM_006250322.3	XM_006250321.3	XP_006250383.1
4	Chimpanzee	XM_016934876.1	XM_016935457.2	XP_016791361.1
5	White-tufted-ear marmoset	XM_017966707.1	XM_008985727.2	XP_008983976.1
6	Cattle	NM_001191309.1	XM_024975966.1	XP_024831736.1
7	Painted turtle	XM_008170470.2	XM_008170470.2	XP_008168692.1
8	Sheep	XM_012187897.2	XM_027975583.1	XP_027831384.1
9	Rhesus monkey	NM_001266640.1	XM_028845179.1	XP_028701012.1
10	Damara mole-rat	XM_010642598.2	XM_010642597.2	XP_010640900.1
11	Chinese tree shrew	XM_014591111.1	XM_006157219.3	XP_014446597.1
12	Water buffalo	XM_006045843.1	XM_025285185.1	XP_006045905.1
13	Domestic ferret	XM_004756776.2	XM_004756776.2	XP_012913885.1
14	Chinese hamster	XM_003508130.3	XM_003508130.4	XP_016833997.1
15	Miniopterus natalensis	XM_016201398.1	XM_016201398.1	XP_016056884.1
16	Egyptian rousette	XM_016151253.1	XM_016151251.1	XP_016006738.1
17	Sooty mangabey	XM_012036298.1	XM_012036296.1	XP_011891692.1
18	Chinese soft-shelled turtle	XM_006135202.2	XM_014579588.2	XP_014435074.1
19	Cheetah	XM_015072881.1	XM_027046229.1	XP_026902031.1
20	Domestic cat	XM_023247428.1	XM_011290838.3	XP_023103194.1
21	Giant panda	XM_011223567.2	XM_011223567.2	XP_011221869.1
22	Green monkey	XM_007989961.1	XM_007989964.1	XP_007988151.1
23	Gray short-tailed opossum	XM_016429466.1	XM_016429466.1	XP_007481609.1
24	Long-tailed chinchilla	XM_005374760.2	XM_005374760.2	XP_005374816.1
25	Naked mole-rat	XM_004853513.3	XM_021265539.1	XP_004853570.1
26	Northern white-cheeked gibbon	XM_012506588.1	XM_030812582.1	XP_030668452.1
27	Przewalski horse	XM_023632779.1	XM_008526929.1	XP_008525153.1
28	Prairie vole	XM_005369606.2	XM_026779836.1	XP_013202436.1
29	Pacific walrus	XM_012562385.1	XM_004400462.2	XP_012417839.1
30	Pig-tailed macaque	XM_011729484.1	XM_011729486.1	XP_011727791.1
31	Sumatran orangutan	XM_009248019.1	XM_002809243.4	XP_024089808.1
32	Wild Bactrian camel	XM_014559995.1	XM_006185561.2	XP_006185623.1
33	Western European hedgehog	XM_007534664.1	XM_007534656.2	XP_007534733.1
34	Weddell seal	XM_006740006.1	XM_031035272.1	XP_006740067.1
35	American beaver	XM_020163460.1	XM_020163460.1	XP_020019049.1
36	Australian saltwater crocodile	XM_019549778.1	XM_019549778.1	XP_019405323.1
37	Koala	XM_020982080.1	XM_020982083.1	XP_020837737.1
38	Olive baboon	XM_017958504.2	XM_003893690.3	XP_017813935.1
39	Zebra fish	NM_001197201.2	XM_001923419.7	XP_001923454.3

**Table 2:** Sites found under positive selection at *AKT3* gene.

REL			MEME		FUBAR
Positive selection sites	dN-dS	Bayes factor	Positive selection sites	*p*	Positive selection sites
25	94.3064	1566.25	250	0.0697201	25
30	94.3515	1630.81	278	0.0129066	30
63	94.3704	1662.42	658	0.001775	63
86	94.1194	1335.47	661	0.00839722	86
			662	0.0755305	250
			664	0.00586727	278
			692	0.00717898	658
			702	0.00524112	661
			714	0.0505746	662
			716	0.0540275	664
					692
					702
					714
					716

**Table 3:** Codon model selection based on modified Bayesian Information Criterion (mBIC) of AKT3 genes.

Classes	Models	Credible	mBIC	*Δ*mBIC	dN/dS (rates in class)		
1	1	0	27660.8		0.08/75		
2	7399	2505	27550.7	110.08	0.04/50		0.17/25
3	845	0	27566.0	-15.26	0.03/21	0.06/29	0.17/25

*N*: number of rate classes included in models; Models: genetic algorithm models; Credible: all the models evaluated by genetic algorithm within 9.21 mBIC unit (the best model has credible values 0.01 or >1); mBIC: modified Bayesian Information Criterion; *Δ*mBIC: mBIC for *N* rate classes compared to *N* − 1 rate classes; dN/dS: maximum likelihood estimates for each rate class.

**Table 4:** List of predicted domains and feature description in *AKT3* of Bos taurus.

S. No.	*Hits by profiles*	AA	Scores	Predicted features			
1	PS50003 PH_Domain	5-107	14.7	DOMAIN	5	107	PH
2	PS50011 PROTEIN_KINASE_DOM	148-405	49.4	DOMAIN	148	405	Protein kinase
				NB_BIND	154	162	ATP
				BINDING	177		ATP
				ACT_SITE	271		Proton acceptor
3	PS51285 AGC_KINASE_CTER	406-479	15.8				
	*Hits by patterns*			Predicted features			
1	PS00107 Protein_Kinase_ATP	154-187					
2	PS00108 Protein_Kinase_ST	267-279		ACT_SITE			Proton acceptor

## Data Availability

The data used to support the findings of this study are included within the article.

## Supplementary Materials

Supplementary Materials
Figure 1: supplementary list of predicted functional partners for AKT3 protein-protein interaction.

## References

[1] Hendrikse J. L., Parsons T. E., Hallgrímsson B. (2007). Evolvability as the proper focus of evolutionary developmental biology. *Evolution & Development*.

[2] Wiens J. J. (2004). What is speciation and how should we study it?. *The American Naturalist*.

[3] Otto S. P. (2009). The evolutionary enigma of sex. *The American Naturalist*.

[4] Manolio T. A., Collins F. S., Cox N. J. (2009). Finding the missing heritability of complex diseases. *Nature*.

[5] Sabeti P. C., Reich D. E., Higgins J. M. (2002). Detecting recent positive selection in the human genome from haplotype structure. *Nature*.

[6] Bionaz M., Loor J. J. (2011). Gene networks driving bovine mammary protein synthesis during the lactation cycle. *Bioinformatics and biology insights*.

[7] Engelman J. A., Luo J., Cantley L. C. (2006). The evolution of phosphatidylinositol 3-kinases as regulators of growth and metabolism. *Nature Reviews Genetics*.

[8] Manning B. D., Cantley L. C. (2007). AKT/PKB signaling: navigating downstream. *Cell*.

[9] Hanada M., Feng J., Hemmings B. A. (2004). Structure, regulation and function of PKB/AKT—a major therapeutic target. *Biochimica et Biophysica Acta (BBA)-Proteins and Proteomics*.

[10] Ullah F., Bhattarai D., Cheng Z. (2018). Comparative analysis of V-AKT murine thymoma viral oncogene homolog 3 (*AKT3*) gene between cow and buffalo reveals substantial differences for mastitis. *BioMed Research International*.

[11] Tsiperson V., Gruber R. C., Goldberg M. F. (2013). Suppression of inflammatory responses during Myelin Oligodendrocyte Glycoprotein–Induced experimental autoimmune encephalomyelitis is regulated by AKT3 signaling. *The Journal of Immunology*.

[12] Yang Z.-Z., Tschopp O., di-Poï N. (2005). Dosage-Dependent Effects of Akt1/Protein Kinase B*α* (PKB*α*) and Akt3/PKB*γ* on Thymus, Skin, and Cardiovascular and Nervous System Development in Mice. *Molecular and Cellular Biology*.

[13] Nakatani K., Thompson D. A., Barthel A. (1999). Up-regulation of Akt3 in estrogen receptor-deficient breast cancers and androgen-independent prostate cancer lines. *Journal of Biological Chemistry*.

[14] Banerji S., Cibulskis K., Rangel-Escareno C. (2012). Sequence analysis of mutations and translocations across breast cancer subtypes. *Nature*.

[15] Tamura K., Stecher G., Peterson D., Filipski A., Kumar S. (2013). MEGA6: molecular evolutionary genetics analysis version 6.0. *Molecular Biology and Evolution*.

[16] Ahmad H. I., Liu G., Jiang X., Liu C., Chong Y., Huarong H. (2017). Adaptive molecular evolution of *MC1R* gene reveals the evidence for positive diversifying selection in indigenous goat populations. *Ecology and Evolution*.

[17] Asif A. R., Awais M., Qadri S., Ahmad H. I., du X. (2017). Positive selection of IL-33 in adaptive immunity of domestic Chinese goats. *Ecology and Evolution*.

[18] Ahmad H. I., Ahmad M. J., Adeel M. M., Asif A. R., du X. (2018). Positive selection drives the evolution of endocrine regulatory bone morphogenetic protein system in mammals. *Oncotarget*.

[19] Ahmad M. J., Ahmad H. I., Adeel M. M. (2019). Evolutionary analysis of makorin ring finger protein 3 reveals positive selection in mammals. *Evolutionary Bioinformatics*.

[20] Poon A. F. Y., Frost S. D. W., Kosakovsky S. L. (2009). Detecting signatures of selection from DNA sequences using Datamonkey. *Bioinformatics for DNA Sequence Analysis*.

[21] Ahmad H. I., Liu G., Jiang X. (2017). Maximum-likelihood approaches reveal signatures of positive selection in BMP15 and GDF9 genes modulating ovarian function in mammalian female fertility. *Ecology and Evolution*.

[22] Bielawski J. P., Yang Z. (2003). Maximum Likelihood Methods for Detecting Adaptive Evolution after Gene Duplication. *Genome Evolution*.

[23] Kelley L. A., Sternberg M. J. E. (2009). Protein structure prediction on the web: a case study using the Phyre server. *Nature Protocols*.

[24] Glaser F., Pupko T., Paz I. (2003). ConSurf: identification of functional regions in proteins by surface-mapping of phylogenetic information. *Bioinformatics*.

[25] Yang J.-R., Liao B.-Y., Zhuang S.-M., Zhang J. (2012). Protein misinteraction avoidance causes highly expressed proteins to evolve slowly. *Proceedings of the National Academy of Sciences*.

[26] Franceschini A., Szklarczyk D., Frankild S. (2012). STRING v9. 1: protein-protein interaction networks, with increased coverage and integration. *Nucleic Acids Research*.

[27] Li H., Zhao X., Wang J., Zong M., Yang H. (2017). Bioinformatics analysis of gene expression profile data to screen key genes involved in pulmonary sarcoidosis. *Gene*.

[28] Quevillon E., Silventoinen V., Pillai S. (2005). InterProScan: protein domains identifier. *Nucleic Acids Research*.

[29] Miyazawa S. (2011). Advantages of a mechanistic codon substitution model for evolutionary analysis of protein-coding sequences. *PLoS One*.

[30] Nielsen R., Yang Z. (1998). Likelihood models for detecting positively selected amino acid sites and applications to the HIV-1 envelope gene. *Genetics*.

[31] Yang Z., Nielsen R., Hasegawa M. (1998). Models of amino acid substitution and applications to mitochondrial protein evolution. *Molecular Biology and Evolution*.

[32] Delport W., Poon A. F. Y., Frost S. D. W., Kosakovsky Pond S. L. (2010). Datamonkey 2010: a suite of phylogenetic analysis tools for evolutionary biology. *Bioinformatics*.

[33] Pond S. L. K., Muse S. V. (2005). HyPhy: hypothesis testing using phylogenies. *Statistical Methods in Molecular Evolution*.

[34] Siepel A., Haussler D. (2004). Combining phylogenetic and hidden Markov models in biosequence analysis. *Journal of Computational Biology*.

[35] Delport W., Scheffler K., Botha G., Gravenor M. B., Muse S. V., Kosakovsky Pond S. L. (2010). CodonTest: modeling amino acid substitution preferences in coding sequences. *PLoS Computational Biology*.

[36] Stanfel L. E. (1996). A New Approach to Clustering the Amino Acid. *Journal of Theoretical Biology*.

[37] Kosiol C., Holmes I., Goldman N. (2007). An empirical codon model for protein sequence evolution. *Molecular Biology and Evolution*.

[38] Posada D., Crandall K. A. (1998). Modeltest: testing the model of DNA substitution. *Bioinformatics*.

[39] Masuko-Hongo K., Berenbaum F., Humbert L., Salvat C., GOLDRING M. B., Thirion S. (2004). Up-regulation of microsomal prostaglandin E synthase 1 in osteoarthritic human cartilage: critical roles of the ERK-1/2 and p38 signaling pathways. *Arthritis & Rheumatism*.

[40] Yu H., Littlewood T., Bennett M. (2015). Akt isoforms in vascular disease. *Vascular Pharmacology*.

[41] Mure H., Hirano S., Tang C. C. (2011). Parkinson's disease tremor-related metabolic network: Characterization, progression, and treatment effects. *NeuroImage*.

[42] Endersby R., Zhu X., Hay N., Ellison D. W., Baker S. J. (2011). Nonredundant functions for Akt isoforms in astrocyte growth and gliomagenesis in an orthotopic transplantation model. *Cancer Research*.

[43] Robertson G. P. (2005). Functional and therapeutic significance of Akt deregulation in malignant melanoma. *Cancer and Metastasis Reviews*.

[44] Wansleben S., Peres J., Hare S., Goding C. R., Prince S. (2014). T-box transcription factors in cancer biology. *Biochimica et Biophysica Acta (BBA)-Reviews on Cancer*.

[45] Ji P., Ma X., Li G. (2015). Developing green purchasing relationships for the manufacturing industry: an evolutionary game theory perspective. *International Journal of Production Economics*.

[46] She B. (2011). Dynamic modeling of the PI3K/Akt signal transduction pathway to dissect the distinct cell fate decisions triggered by PI3K/Akt signaling in hematopoietic system.

[47] Axelsson E., Webster M. T., Smith N. G., Burt D. W., Ellegren H. (2005). Comparison of the chicken and turkey genomes reveals a higher rate of nucleotide divergence on microchromosomes than macrochromosomes. *Genome Research*.

[48] Auclair S., Rossetti R., Meslin C. (2013). Positive selection in bone morphogenetic protein 15 targets a natural mutation associated with primary ovarian insufficiency in human. *PLoS One*.

[49] Persani L., Rossetti R., Di Pasquale E., Cacciatore C., Fabre S. (2014). The fundamental role of *bone morphogenetic protein 15* in ovarian function and its involvement in female fertility disorders. *Human Reproduction Update*.

[50] Bergström T., Gyllensten U. (1995). Evolution of Mhc class II polymorphism: the rise and fall of class II gene function in primates. *Immunological Reviews*.

[51] Kan Z., Jaiswal B. S., Stinson J. (2010). Diverse somatic mutation patterns and pathway alterations in human cancers. *Nature*.

[52] Scannell D. R., Zill O. A., Rokas A. (2011). The awesome power of yeast evolutionary genetics: new genome sequences and strain resources for theSaccharomyces sensu strictoGenus. *G3: Genes, Genomes, Genetics*.

[53] Chin Y. R., Yoshida T., Marusyk A., Beck A. H., Polyak K., Toker A. (2014). Targeting Akt3 signaling in triple-negative breast cancer. *Cancer Research*.

[54] Corum D. G., Tsichlis P. N., Muise-Helmericks R. C. (2013). AKT3 controls mitochondrial biogenesis and autophagy *via* regulation of the major nuclear export protein CRM-1. *The FASEB Journal*.

[55] Ding L., Ley T. J., Larson D. E. (2012). Clonal evolution in relapsed acute myeloid leukaemia revealed by whole- genome sequencing. *Nature*.

[56] Dümmler B. (2007). *Physiological roles of PKB isoforms in development, growth and glucose metabolism*.

[57] Easton R. M., Cho H., Roovers K. (2005). Role for Akt3/protein kinase Bgamma in attainment of normal brain size. *Molecular and Cellular Biology*.

[58] Khan M. (2013). *Effects of Prepartum Dietary Energy and Lipid Supplementation on Hepatic Transcriptome Profiles in Dairy Cows during the Transition Period*.

[59] Madhunapantula S. V., Robertson G. P. (2009). The PTEN–AKT3 signaling cascade as a therapeutic target in melanoma. *Pigment Cell & Melanoma Research*.

[60] Turner K. M., Sun Y., Ji P. (2015). Genomically amplified Akt3 activates DNA repair pathway and promotes glioma progression. *Proceedings of the National Academy of Sciences*.

