# Supplementary material for: Adaptive Molecular Evolution of AKT3 Gene for Positive Diversifying Selection in Mammals
Source: Biomed Res Int. 2020 May 19;2020:2584627. doi: 10.1155/2020/2584627 (PMC7256775; doi:10.1155/2020/2584627)
Supplement: Supplementary Materials — Figure 1: supplementary list of predicted functional partners for AKT3 protein-protein interaction. [file 2584627.f1.docx]

**
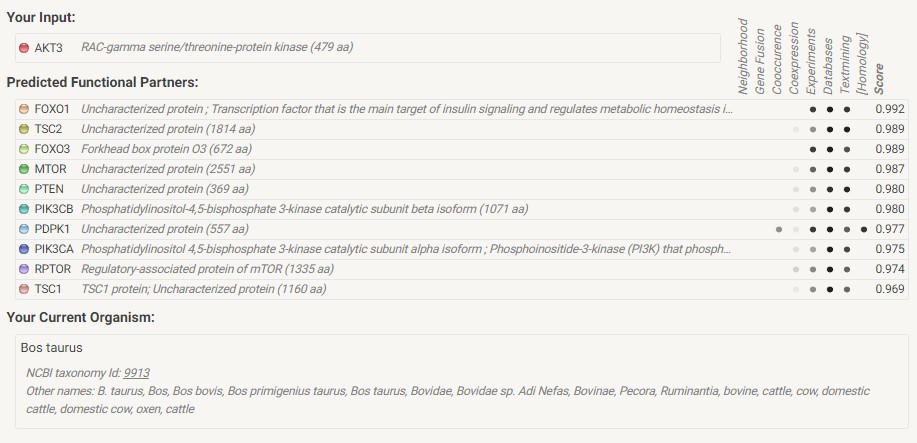
**

**Figure-1**. Supplementary List of predicted functional partners for *AKT3* protein-protein Interaction
